# Color Dependence Analysis in a CNN-Based Computer-Aided Diagnosis System for Middle and External Ear Diseases

**DOI:** 10.3390/diagnostics12040917

**Published:** 2022-04-07

**Authors:** Michelle Viscaino, Matias Talamilla, Juan Cristóbal Maass, Pablo Henríquez, Paul H. Délano, Cecilia Auat Cheein, Fernando Auat Cheein

**Affiliations:** 1 Department of Electronic Engineering, Universidad Técnica Federico Santa María, Valparaíso 2390382, Chile; michelle.viscaino@sansano.usm.cl; 2 Advanced Center of Electrical and Electronic Engineering, Valparaíso 2390136, Chile; pdelano@med.uchile.cl; 3 Interdisciplinary Program of Physiology and Biophysics, Institute of Biomedical Sciences (ICBM), Faculty of Medicine, University of Chile, Santiago 8320328, Chile; matiastalamilla@ug.uchile.cl (M.T.); juanmaass@uchile.cl (J.C.M.); 4 Department of Otolaryngology, Hospital Clínico Universidad de Chile, Faculty of Medicine, University of Chile, Santiago 8320328, Chile; pablohenriquezc@ug.uchile.cl; 5 Unit of Otolaryngology, Department of Surgery, Clínica Alemana de Santiago, Facultad de Medicina Clínica Alemana-Universidad del Desarrollo, Santiago 0323142, Chile; 6 Medical Sciences Doctorate Program, Postgraduate School, Faculty of Medicine, University of Chile, Santiago 8320328, Chile; 7 Department of Neuroscience, Faculty of Medicine, University of Chile, Santiago 8320328, Chile; 8 Facultad de Ciencias Médicas, Universidad Nacional de Santiago del Estero, Santiago del Estero 4200, Argentina; ceciauat@gmail.com

**Keywords:** otology, artificial intelligence, middle and external ear, deep learning, convolutional neural network

## Abstract

Artificial intelligence-assisted otologic diagnosis has been of growing interest in the scientific community, where middle and external ear disorders are the most frequent diseases in daily ENT practice. There are some efforts focused on reducing medical errors and enhancing physician capabilities using conventional artificial vision systems. However, approaches with multispectral analysis have not yet been addressed. Tissues of the tympanic membrane possess optical properties that define their characteristics in specific light spectra. This work explores color wavelengths dependence in a model that classifies four middle and external ear conditions: normal, chronic otitis media, otitis media with effusion, and earwax plug. The model is constructed under a computer-aided diagnosis system that uses a convolutional neural network architecture. We trained several models using different single-channel images by taking each color wavelength separately. The results showed that a single green channel model achieves the best overall performance in terms of accuracy (92%), sensitivity (85%), specificity (95%), precision (86%), and F1-score (85%). Our findings can be a suitable alternative for artificial intelligence diagnosis systems compared to the 50% of overall misdiagnosis of a non-specialist physician.

## 1. Introduction

Otolaryngology is a medical-surgical discipline that includes the prevention, diagnosis, and treatment of different structures of the ear, nasal and paranasal cavities, pharynx, larynx, trachea, head and neck [1]. It is estimated that between 10–20% of general practitioner consultations are for ENT (ear, nose, and throat) complaints. In children, this rate rises to around 50%. More significantly, ENT referrals constitute the third largest group of patients referred to hospital specialists, where middle ear disorders are the most frequent diseases in daily ENT practice [2]. Of particular note is chronic otitis media, the leading cause of conductive hearing loss worldwide, affecting 65 to 330 million people, specially in low-income countries [3].

According to the Institute of Medicine of the National Academies of Science, Engineering, and Medicine from the US, nearly all patients will experience a diagnostic error in their life [4]. In the US alone, approximately between 50,000–100,000 patients potentially may die each year due to medical errors. Although otolaryngology has always been regarded as a safe specialty due to the low morbidity and mortality rates, it is estimated that the diagnostic error of non-specialist physicians is around 50%. Such percentage decreases to close 30% when a specialist makes the diagnosis, but both values are still high [5]. The latter is detrimental to the proper implementation of treatments, increases health costs, and could lead to severe complications for the patient. There is a clear need to strengthen diagnosis and consequent otolaryngological treatment in the health system. Due to the advent of advanced processing techniques and the large volume of digital data available today, new technology to assist medical diagnosis has been generated as an increasing tendency to reduce diagnostic error in the last few years [6]. Some studies [7,8,9] have demonstrated the benefit of using artificial intelligence (AI) to improve and diagnose safety for ENT pathologies of various types by achieving a diagnostic performance of over 90%.

Otological analysis has recently attracted the attention from the scientific community to generate new ear databases and to apply deep learning techniques [10,11]. However, this analysis has mainly been carried out using images obtained with white light during the external and middle ear examination by otoscopy or otoendoscopy. But tissues possess optical properties that define their characteristics in specific light spectra. For example, hemoglobin preferentially absorbs the light spectrum between 500 nm and 600 nm, whereas water, lipids, or collagen predominantly absorbs infrared light spectra [12]. The tympanic membrane and tympanic cavity also have different optical absorption and penetration properties. For example, the tympanic membrane has an absorption pick in shorter wavelengths of the visible light spectrum (400–600 nm), while for wavelengths shifted to the red or infrared (greater than 1000 nm), absorption is weak, having this chromatic spectrum greater penetration through the tympanic membrane to the tympanic cavity [13].

The state of the art of spectral analysis for diagnosing middle ear pathologies is limited and recent. One study published in 2015 [14] showed that differential absorption at the multiple wavelengths provides a measure of biochemical and morphological information of tympanic membrane. The study was conducted using a modified multi-wavelength narrow-band otoscope in a limited cohort of five patients. More recently, a study [15] developed an analysis of otoscopy images acquired in visible spectra and concluded that the blue and green channels have an absorption pick for hemoglobin, highlighting vascular structures. In contrast, the red channel has a high penetrance to the tympanic membrane, allowing a better visualization of the structures inside the tympanic cavity. Another study [9] identified the presence of effusions in the middle ear using machine learning algorithms. The most important contribution was developing an otoscope that visualizes middle ear structures and fluid in the shortwave infrared region. Although such an otoscope is not commercially available, it encourages the development of new technology considering a multispectral approach.

Our previous work [8] proposed a scheme for otological diagnosis using RGB (red, green, and blue) images from the external auditory canal. The model achieved high performance for nine ear pathologies (over 90%). However, the diagnosis of some relevant pathologies such as chronic otitis media (COM) or otitis media with effusion (OME) that arises from specific characteristics such as vascularization of the membrane (COM) or the presence of fluid (OME), can be analyzed in a particular spectral band to obtain more helpful information for the physician. In this work, we evaluate the dependence of different color wavelengths in the performance of a computer-aided diagnosis system based on convolutional neural networks. First, we preprocessed the data to discard useless information and implemented a novel video summarization technique to reduce redundant information. Next, we trained a customized convolutional neural network from scratch to predict four possible diagnoses: normal ear, COM, OME, and earwax plug. Finally, we evaluate the relevant regions as a class activation heatmap employed by the model to perform the prediction.

## 2. Materials and Methods

This section describes the ear imagery database and data preprocessing in detail. Also, we present the architecture based on a convolutional neural network as a predictor of the four ear conditions under study. Finally, this section shows the metrics used to evaluate the performance of the proposed models.

### 2.1. Ear Imagery Database

Retrospective analysis of images from video otoscopy was performed in patients who consulted at the Otolaryngology Departament of the Clinical Hospital of the University of Chile (HCUCH) between 2018 and 2019. A total of 22,000 images of the eardrum from 195 patients were used as the database for this work. The images were extracted from 200 video otoscopies acquired by ENT specialists using a digital otoscope DE500 Firefly. The video files were recorded at 20 FPS (frames per second) with a 640 × 480 pixels resolution.

**Figure 1:**
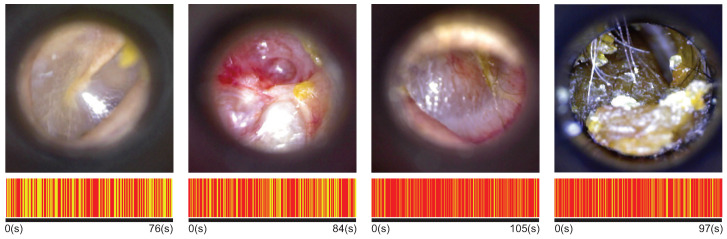
Upper row: eardrum imaging for each ear condition. From left to right: normal, chronic otitis media, otitis media with effusion, and earwax plug. Lower row: representation of the frames in a video, the yellow lines are those selected as keyframes.

The ENT specialists of HCUCH performed at least two otoscopy examinations per patient (left and right ear). They recorded up to three diagnoses for each ear. Our analysis included those video otoscopies of patients with only one diagnosis related to four ear conditions: normal, chronic otitis media (COM), otitis media with effusion (OME), and earwax plug. The first row in Figure 1 shows an example of each ear condition.

The study was approved by the Scientific or Research Ethics Committee of the Clinical Hospital from University of Chile (approval number 65 996/18). All patients gave written informed consent, and all procedures were conducted following national regulations and the declaration of Helsinki, revision 2013.

### 2.2. Data Pre-Processing

We propose an image-level analysis of the frames extracted from each video otoscopy. Some frames showed neither the ear canal nor the eardrum because the otoscopy recording started or ended at a different location than the ear. In addition, blurred frames occurred during acquisition due to camera movement (i.e., digital otoscope) or camera out-of-focus, which the physician manually adjusted during the examination. We discarded all those undesirable frames by first separating the images that effectively show the tympanic membrane and then applying an algorithm to detect the level of blur in the image (considering only uniform blurring).

#### 2.2.1. Image Domain Analysis

Several images acquired during otoscopy do not show the tympanic membrane or ear canal. To avoid confusing the network by introducing useless information, we discard all images outside the domain of interest.

Useless images were acquired at the beginning or the end of the otoscopy due to the nature of the acquisition procedure. On the one hand, the physician starts the recording before inserting the specula of the digital otoscope into the patient’s ear to ensure that the camera is in focus (manual focus). On the other hand, the recording ends once the specula have been removed from the patient’s ear producing unnecessary frames. Assuming that the central frame of the video shows the tympanic membrane and (or) the ear canal [8], we calculate the histogram of such a frame and compare it to each of the frames in the video using the Kullback-Leibler divergence score. If the score falls below a certain threshold, the frame is discarded.

#### 2.2.2. Blurring Detector

To evaluate the blurriness in the image, we employ the Variance of the Laplacian method as presented in [7]. Such a method computes the variance of convolving a single image channel with the Laplacian Kernel. If the variance falls below a predefined threshold, the image is considered blurred, and we discarded it. The Variance of the Laplacian method is based on the fact that the Laplacian operator highlights regions of an image containing rapid intensity changes (i.e., edges). Therefore, an image with a low variance is considered blurred.

#### 2.2.3. Keyframes Selection

To avoid the redundancy of the videos recorded, we implement a video summarization technique based on Principal Component Analysis (PCA) and a clustering algorithm (e.g., K-means). As a result, we obtain a subset of the most informative frames (keyframes) from a video otoscopy to feed the deep neural network.

Once all blurred and out-of-domain frames were discarded, PCA was applied to find those components that describe a whole image in the video. The first 20 components were selected as principal components because, in our case, they explain 80% of the variance of data. Also, the dimension of the new feature space allows decreasing the computational cost of the process. Finally, we applied k-means to select N keyframes. The clustering algorithm assigned each frame (represented by the previously obtained principal components) to a specific cluster considering the smallest distance (e.g., Euclidean distance). We select only one image per cluster, considering the one whose Euclidean distance is the smallest.

The duration of the videos ranged from 30 s until 120 s. The shorter videos belong to the cerumen plug cases because the physician can only observe the earwax plug in the tympanic cavity (except in partial earwax plug cases). In more complex conditions, the physician performs a more extended examination to inspect the entire tympanic membrane and gather as much information as possible. There are more data (frames) of certain ear conditions in this sense. To avoid bias in the results due to an unbalanced database, we apply the keyframe selection algorithm with k = N = 100 and use the same amount of videos for each class. The bottom row of Figure 1 represents the selection of keyframes for different video durations depending on the class. The lines in yellow represent those frames selected.

### 2.3. Computer-Aided Diagnosis (CAD) System

Our proposal is implemented under the configuration of a CAD system. An image of the tympanic membrane feeds a convolutional neural network that predicts four possible diagnoses: normal ear, chronic otitis media, otitis media with effusion, or an earwax plug. The network was previously trained on our otoscopy ear database. The spectral analysis of the input data to the system and the neural network’s architecture are discussed below.

#### 2.3.1. Image Spectral Analysis

The database contains images in the RGB color space. That is, each pixel in the image is composed of three values representing the light intensity in the three channels: red (618–780 nm), green (497–570 nm), and blue (427–476 nm). With the aim to analyze the color dependence in CNN performance, we process the images to obtain single-channel information.

**Figure 2:**
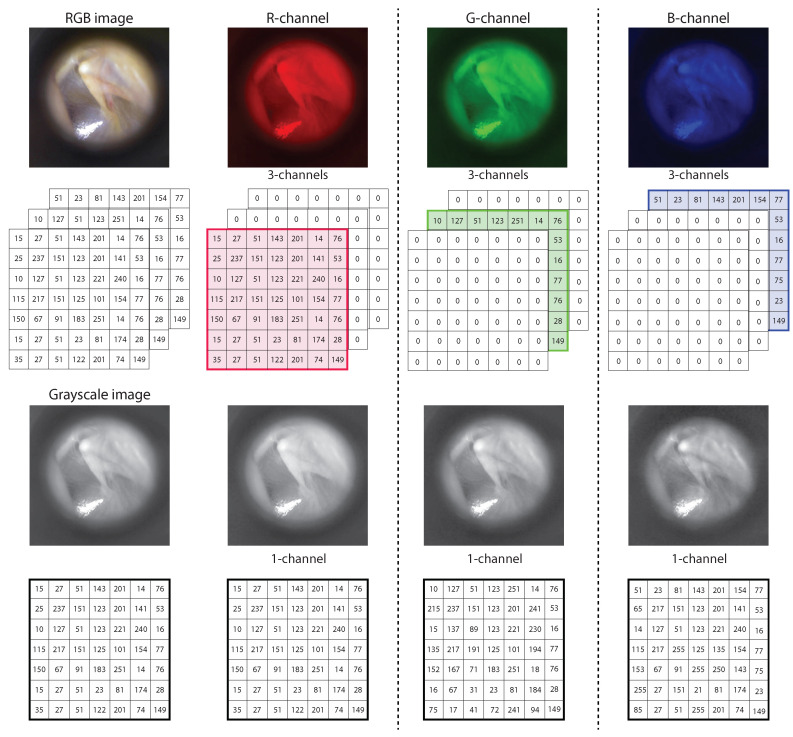
Matrix representation of a digital image: (upper row, from left to right) image in the RGB color space, 3-channel image with the non-zero red channel, 3-channel image with the non-zero green channel, 3-channel image with the non-zero blue channel; (lower row, from left to right) weighted linear combination grayscale image, red channel grayscale image, green channel grayscale image, blue channel grayscale image.

The first approach is to keep images of three channels but with two equal to zero, as shown in the top row of Figure 2. The network receives all three channels as input data. However, only the non-zero channel will provide the network with information. The next step was to work with single-channel images (grayscale images) with intensity values of each channel, as shown in the bottom row of Figure 2. In this case, the network is set to work with single-channel images. Finally, we also use grayscale images converted from the RGB images as a weighted linear combination of the three channels. Each pixel (i,j) of the grayscale image is calculated as:

(1) $$g_{i,j}=0.2989\cdot R_{i,j}+0.5870\cdot G_{i,j}+0.1140\cdot B_{i,j}$$

#### 2.3.2. Convolutional Neural Network

Our approach is based on the VGG-16 neural network [16], which has a simple configuration with a quite deep architecture (about 138 million trainable parameters). In addition, the reduced number of hyperparameters compared to other architectures makes the VGG-16 network a practical solution for training from scratch.

**Figure 3:**
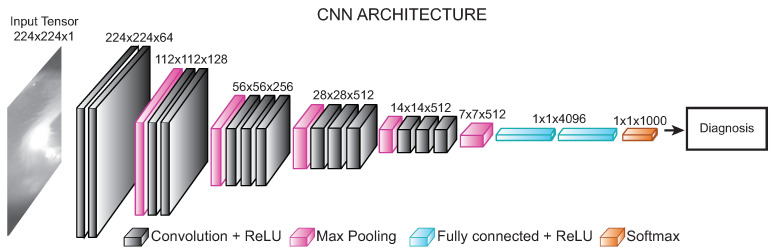
Convolutional neural network architecture for image classification.

The network was adapted to work with a single image channel with an input tensor of 224×224×1 or 224×224×3 depending on the input data. The architecture, as well as the output dimension of each layer, is shown in Figure 3. The network maintains a consistent layout with convolution layers of a 3×3 kernel-sized filter (always of stride 1, and the same padding), maximum pooling layer of a 2×2 kernel-sized filter, and stride 2. In the end, it has two fully connected layers (FC) followed by a softmax layer for the output (prediction).

First convolutional layers extract low-level features such as edges, corners, or lines from the image. The last convolutional layers recognize structures and larger shapes in the input data. Each convolutional layer generates a feature map $X_{i}^{l-1}$ feeding to the next layer by convolving the input with the learnable kernels $k_{ij}^{l}$ and a trainable bias parameter $b_{j}^{l}$ and passing through an activation function g(.) as shown in Equations (2) and (3).

(2) $$Z_{j}^{l}=\sum_{i\in M_{j}}X_{i}^{l-1}*k_{ij}^{l}+b_{j}^{l}$$

(3) $$X_{i}^{l}=g\left(Z_{j}^{l}\right)$$

where, *l* represents the number of layer, *j* de number of neuron inside the layer, and *i* de *ith* element in the input map $M_{j}$. In our proposal, the activation function g(.) is a Rectified Linear Units (ReLU) [17].

Pooling layer reduces the number of computational nodes and prevents overfitting. There are not learnable parameters just realize a downsampling operation using average or maximum methods. This layer is include to provide a summary of the local distinctive features.

Fully connected layer flattens the input data, typically the output of a convolutional or pooling layer, and performs the same operations given in Equations (2) and (3). The information moves in one direction to a final softmax activation function. Based on Luce’s choice axiom, softmax normalizes the output to a probability distribution over the predicted output class [18].

### 2.4. Evaluation Metrics

We use a confusion matrix, which is a tool often used to describe the performance of a classification model. The matrix is generally defined for a binary approach with a positive and negative class (e.g., the patient has or does not have a particular disease). However, it can be extended to a multi-class approach by using the One-vs-All method [19]. The confusion matrix is formed from four possible outcomes: true positive (TP) and true negative (TN) are all instances where the model correctly predicts the positive class or negative class, respectively; false positive (FP) and false negative (FN) are all those instances incorrectly predicted by the model as the positive class or negative class, respectively.

From the confusion matrix, other metrics are computed. The most widely used metric is accuracy formulated in Equation (4), which represents the overall effectiveness of the classifier. However, this metric is strongly dependent on the data distribution and can be biased in imbalanced datasets [20]. Other metrics can be helpful in medical applications, such as sensitivity, see Equation (5), that quantifies the capability of the model to avoid false negatives. A high value of specificity described by Equation (6) is desirable in the model, because it implies that the model is able to identify those true negative outcomes.

(4) $${Accuracy}_{M}=\frac{\sum_{i=1}^{c}\frac{{TP}_{i}+{TN}_{i}}{{TP}_{i}+{FN}_{i}+{FP}_{i}+{TN}_{i}}}{c}$$

(5) $${Sensitivity/recall}_{M}=\frac{\sum_{i=1}^{c}\frac{{TP}_{i}}{{TP}_{i}+{FN}_{i}}}{c}$$

(6) $${Specificity}_{M}=\frac{\sum_{i=1}^{c}\frac{{TN}_{i}}{{TN}_{i}+{FP}_{i}}}{c}$$

(7) $${Precision/PPV}_{M}=\frac{\sum_{i=1}^{c}\frac{{TP}_{i}}{{TP}_{i}+{FP}_{i}}}{c}$$

(8) $$F1-score=\frac{2\cdot precision\cdot recall}{precision+recall}$$

where *c* represents the number of classes (i=1,…,4). We also used the method One vs. All to plot the receiver operating characteristic (ROC) curve. The horizontal axis represents the False positive rate (1-specificity) in such a curve. In contrast, the vertical axis represents the True positive rate (or sensitivity). A suitable classifier is expected to have the ROC curve as close to the upper left corner as possible in the graphical space.

## 3. Results

This section presents the experimental setup and configuration, the performance of individual networks using different channels, and a graph of the relevant area used for each classifier to make the diagnosis prediction.

### 3.1. Experimental Setup and Configuration

We implemented the proposed scheme using the TensorFlow framework with Python programming language. For training, we used an Intel 2.9-GHz CPU and NVIDIA GeForce GTX1080 GPU.

After the keyframes selection stage, the ear imagery database resulted is balanced and contains 22,000 images from four ear conditions: normal (5500 samples), OMC (5500 amples), OME (5500 samples), and earwax plug (5500 samples). The database was randomly partitioned into training/validation and testing sets with no overlap. We employed 500 samples of each class for testing. The remaining 5000 images were used for training and validation in a partition of 80% and 20%, respectively. The results reported below are derived after an average of 10 trials with the respective dataset partitioning.

The input tensor of every single model receives an image of 224×224. Therefore, all images were resized before feeding the neural network. In addition, a real-time data augmentation technique, available in Keras, was implemented to prevent the model from overfitting. Such an option allows generated new samples per batch during the training by performing rotations, zoom-in, zoom-out, and horizontal/vertical flips on the original data. The hyperparameters were set as given in [16], using Adam stochastic optimization [21] with a learning rate of $10^{-5}$. The batch size was 32, with 100 epochs during the training process.

### 3.2. Evaluation of Single-Channel CNN Model

**Figure 4:**
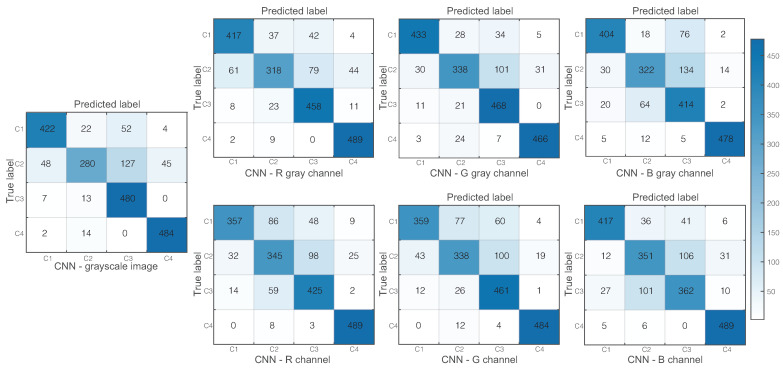
Confusion matrix for each trained model using: weighted linear combination grayscale images, red channel grayscale images, green channel grayscale images, blue channel grayscale images, 3-channel images with the non-zero red channel, 3-channel images with the non-zero green channel, 3-channel images with the non-zero blue channel. C1: normal, C2: chronic otitis media, C3: otitis media with effusion, and C4: earwax plug.

We compute the confusion matrices for the seven models implemented as shown in Figure 4. One model was trained with grayscale images converted from the RGB images. The other three models were trained with grayscale images from the R, G, and B channels, respectively. Finally, the remaining three models were trained with three-channel images, but only one channel enabled (i.e., non-zero) R, G, and B.

**Figure 5:**
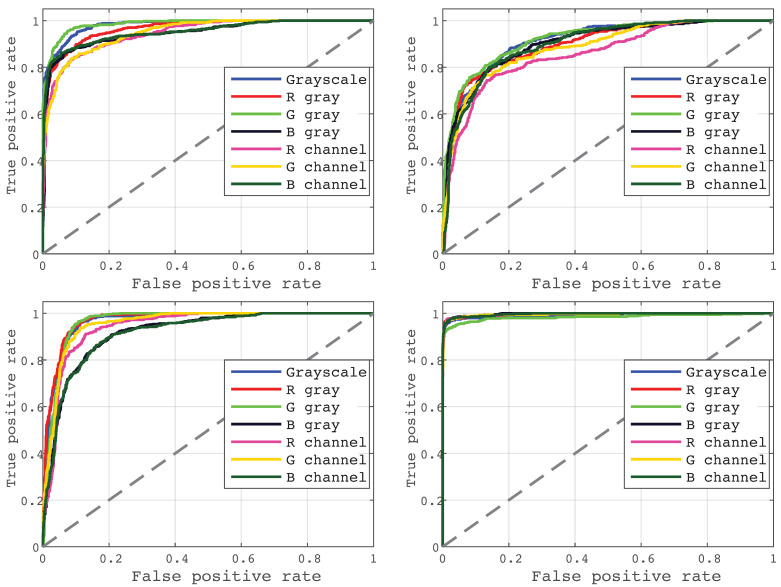
ROC curves generated by all models on the testing set for: (upper row, from left to right) normal ear, chronic otitis media; (lower row, from left to right) otitis media with effusion, and earwax plug.

We have plotted ROC curves per each ear condition from seven models, as shown in Figure 5. As can be seen, all models can give a promising diagnosis on all four models. However, the performance of the classifiers is better at predicting earwax plug conditions. In contrast, predicting COM is more challenging.

**Figure 6:**
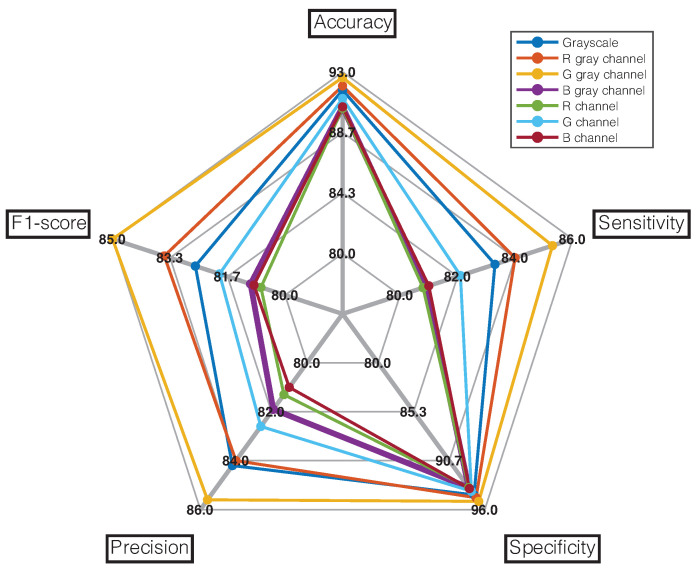
Radial graph showing overall performance comparison of all implemented models. The evaluation was conducted on the testing set.

Using the metrics established in Equations (4)–(8), we evaluated the results of all models to find the best classifier that fits the data in the diagnosis of the four ear conditions under study, as shown in Figure 6. The radial chart shows that the model that achieved the best performance in terms of accuracy (92.6%), sensitivity (85.25%), specificity (95.1%), precision (85.6%), and F1-score (85%) was the one trained with grayscale green channel images. We also show the metrics for each ear condition, and these are summarized in Table 1. The highest values of each evaluation metric per pathology were highlighted in bold. It can be seen that the model trained with grayscale images of the green channel is still adequate for predicting normal ear and COM cases. However, a more accurate prediction of OME cases is obtained when working with the red channel as well as the accurate prediction of earwax plug cases is obtained from the blue channel.

**Figure 7:**
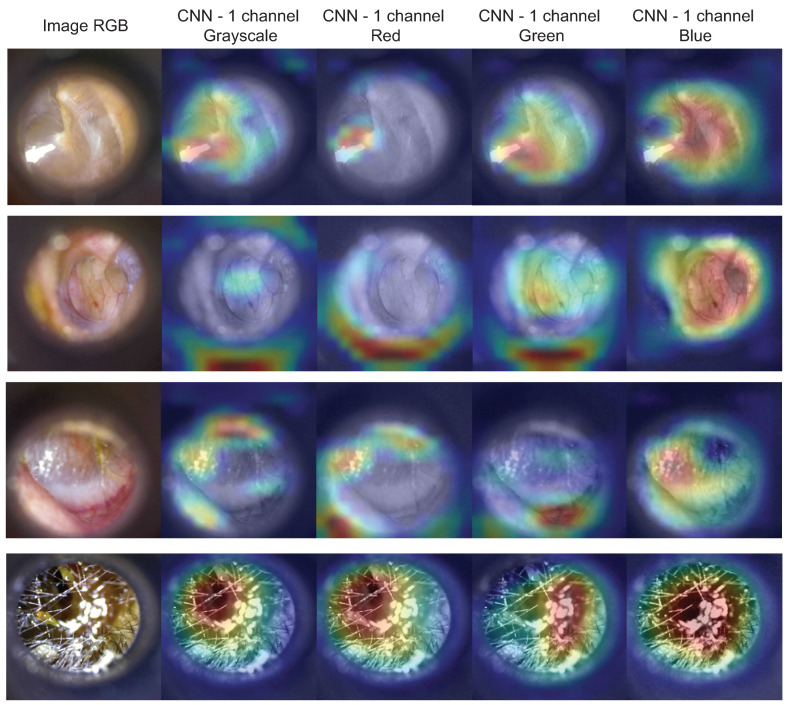
Visual representation of class activation heatmap obtained from the last group of convolutional neural networks using Grad-CAM, overlapped with an original image: (per row, from top to bottom) normal ear, chronic otitis media, otitis media with effusion, and earwax plug.

We employed Gradient-weighted Class Activation Mapping (Grad-CAM) [22] to visualize the regions of input data that are relevant for predictions from the models. The class activation heatmap for each CNN model was extracted from the last group of convolutional layers. The results shown in Figure 7 reveal the model’s most important regions to be considered to perform the prediction. Visually, the blue channel model correctly considers the tympanic membrane region for the prediction. In the case of earwax plug, the wax region is always correctly considered for prediction in all models.

## 4. Discussion

There is a clear need to strengthen the diagnosis and consequent otolaryngological treatment in health systems. The accurate diagnosis of middle and external ear diseases is challenging due to similar signs and symptoms between several pathologies. In addition, for almost all cases is necessary an inspection using an otoscope, a tool that has remained unchanged for, over a half-century. The inspection-based diagnosis will depend on the expertise of the examiner.

Multispectral imaging analysis has several advantages over standard otoscopy, including increased image contrast, clear visualization of middle ear elements, better assessment of tympanic membrane vascularity, and improved demarcation of critical morphological structures (e.g., the malleus and the promontory). Although there are no commercial otoscopes available that acquire images in different spectral bands in addition to RGB, the research results of the few works [9,14,15] in state of the art encourage the development of new technologies considering a multispectral approach. In this work, we explored the dependence of three different color wavelengths, including red, green, and blue channels, in the performance of a CNN-based model to predict the diagnosis of four ear conditions. All models could predict the diagnosis with higher accuracy than the non-specialist physician (50%). However, COM is more challenging to predict, as evidenced by the ROC curve presented in Figure 5, which sensitivity is also the lowest among the four conditions (70%) –followed by normal (84.4%), OME (96%), and earwax plug (97.8%). In fact, all models present high performance when predicting cases of earwax plug, as shown in Table 1.

The findings in this work encourage an analysis in which more pathologies that a physician is confronted with in daily practice can be included, as well as an images analysis in non-visible spectra such as infrared or ultraviolet.

## 5. Conclusions

Middle and external ear diseases with conductive hearing loss are frequent medical consult of general practitioners and ENT specialists. However, misdiagnosis reaches rates around 30–50%. We evaluate the dependence of different color wavelengths in the performance of a computer-aided diagnosis system based on convolutional neural networks. The results showed that the model trained on green channel grayscale images outperformed others models in terms of accuracy (92%), sensitivity (85%), specificity (95%), precision 12 (86%), and F1-score (85%). In addition, our findings showed that the model trained with grayscale images of the green channel is still adequate for predicting normal ear and chronic otitis media cases. However, a more accurate prediction for otitis media with effusion cases is obtained when working with the red channel. Whereas, the accurate prediction of earwax plug cases is obtained from the blue channel. Also, the system could assist in different areas of medicine such as primary health care consultation, emergency rooms, clinics, and hospitals that demonstrate the usefulness and versatility of the method, especially for the general practitioner and the ENT specialist in case of diagnostic doubt. More studies are needed to associate the improvement in diagnostic performance with the establishment of clinical outcomes, such as early initiation of treatment, reduction in adverse effects and complications, or clinical prognosis.

## Figures and Tables

**Table 1:** System performance comparison per class (diagnosis) using testing set.

Metric	Normales	COM	OME	Earwax Plug	Model
Accuracy	93.3	86.6	90.1	96.8	Grayscale
	92.3	87.4	**91.9**	96.5	R gray channel
	**94.5**	**88.3**	91.3	96.5	G gray channel
	92.5	86.4	85.0	**98.0**	B gray channel
	90.6	84.6	88.8	97.7	R channel
	90.2	86.2	89.9	**98.0**	G channel
	93.7	85.4	85.8	97.1	B channel
Sensitivity	**84.4**	56.0	**96.0**	96.8	Grayscale
	83.4	63.3	91.6	91.9	R gray channel
	86.6	67.6	93.6	93.2	G gray channel
	80.8	64.4	82.8	95.6	B gray channel
	71.4	69.0	85.0	**97.8**	R channel
	71.8	67.6	92.2	96.8	G channel
	83.4	**70.2**	72.4	**97.8**	B channel
Specificity	96.2	**96.7**	88.1	96.7	Grayscale
	95.3	95.4	**91.9**	96.1	R gray channel
	**97.1**	95.1	90.5	97.6	G gray channel
	96.3	93.7	85.7	**98.8**	B gray channel
	96.9	89.8	90.1	97.6	R channel
	96.3	92.3	89.1	98.4	G channel
	**97.1**	90.5	90.2	96.9	B channel
Precision	88.1	**85.1**	72.8	90.8	Grayscale
	85.5	82.2	**79.1**	89.2	R gray channel
	**90.8**	82.2	76.7	92.8	G gray channel
	88.0	77.4	65.8	**96.4**	B gray channel
	88.6	69.3	74.0	93.1	R channel
	86.7	74.6	73.8	95.3	G channel
	90.5	71.1	71.1	91.2	B channel
F1-score	86.2	67.6	82.8	93.7	Grayscale
	84.4	71.5	**84.9**	93.3	R gray channel
	**88.6**	**74.2**	84.3	93.0	G gray channel
	84.3	70.3	73.3	**96.0**	B gray channel
	79.1	69.1	79.1	95.4	R channel
	78.6	70.9	82.0	**96.0**	G channel
	86.8	70.6	71.8	94.4	B channel

## Data Availability

Data and materials used in this study are available upon reasonable request to the corresponding author and under a collaboration agreement.

## Abbreviations

The following abbreviations are used in this manuscript:

ENT	Ear, nose, and throat
CNN	Convolutional neural network
RGB	Red, green, blue
COM	Chronic otitis media
OME	Otitis media with efussion
HCUCH	Clinical Hospital of the University of Chile
PCA	Principal component analysis
fps	frames per second
CAD	Computer-aided diagnosis
FC	Fully connected layer
ReLU	Rectified linear unit
